# Predictors of Pregnancy Termination among Young Women in Ghana: Empirical Evidence from the 2014 Demographic and Health Survey Data

**DOI:** 10.3390/healthcare9060705

**Published:** 2021-06-10

**Authors:** Bright Opoku Ahinkorah, Abdul-Aziz Seidu, John Elvis Hagan, Anita Gracious Archer, Eugene Budu, Faustina Adoboi, Thomas Schack

**Affiliations:** 1 School of Public Health, Faculty of Health, University of Technology Sydney, Sydney 2007, Australia; bright.o.ahinkorah@student.uts.edu.au; 2 Department of Population and Health, University of Cape Coast, Cape Coast 0494, Ghana; abdul-aziz.seidu@stu.ucc.edu.gh (A.-A.S.); eugene.budu@stu.ucc.edu.gh (E.B.); 3 College of Public Health, Medical and Veterinary Sciences, James Cook University, Townsville 4811, Australia; 4 Department of Health, Physical Education, and Recreation, University of Cape Coast, Cape Coast 0494, Ghana; 5 Neurocognition and Action-Biomechanics-Research Group, Faculty of Psychology and Sports Science, Bielefeld University, Postfach 1001 31, 33501 Bielefeld, Germany; thomas.schack@uni-bielefeld.de; 6 School of Nursing and Midwifery, University of Health and Allied Sciences, Sokode-Lokoe PMB 31, Ho 342-0041, Ghana; nanaakuaadufa@gmail.com; 7 Cape Coast Nursing and Midwifery Training College, Cape Coast 729, Ghana; faustinaadoboi@yahoo.com

**Keywords:** Ghana, induced abortion, miscarriage, pregnancy termination, stillbirth, young women

## Abstract

Pregnancy termination remains a delicate and contentious reproductive health issue because of a variety of political, economic, religious, and social reasons. The present study examined the associations between demographic and socio-economic factors and pregnancy termination among young Ghanaian women. This study used data from the 2014 Demographic and Health Survey of Ghana. A sample size of 2114 young women (15–24 years) was considered for the study. Both descriptive (frequency, percentages, and chi-square tests) and inferential (binary logistic regression) analyses were carried out in this study. Statistical significance was pegged at *p* < 0.05. Young women aged 20–24 were more likely to have a pregnancy terminated compared to those aged 15–19 (AOR = 3.81, CI = 2.62–5.54). The likelihood of having a pregnancy terminated was high among young women who were working compared to those who were not working (AOR = 1.60, CI = 1.19–2.14). Young women who had their first sex at the age of 20–24 (AOR = 0.19, CI = 0.10–0.39) and those whose first sex occurred at first union (AOR = 0.57, CI = 0.34–0.96) had lower odds of having a pregnancy terminated compared to those whose first sex happened when they were less than 15 years. Young women with parity of three or more had the lowest odds of having a pregnancy terminated compared to those with no births (AOR = 0.39, CI = 0.21–0.75). The likelihood of pregnancy termination was lower among young women who lived in rural areas (AOR = 0.65, CI = 0.46–0.92) and those in the Upper East region (AOR = 0.18, CI = 0.08–0.39). The findings indicate the importance of socio-demographic factors in pregnancy termination among young women in Ghana. Government and non-governmental organizations in Ghana should help develop programs (e.g., sexuality education) and strategies (e.g., regular sensitization programs) that reduce unintended pregnancies which often result in pregnancy termination. These programs and strategies should include easy access to contraceptives and comprehensive sexual and reproductive health education. These interventions should be designed considering the socio-demographic characteristics of young women. Such interventions will help to achieve Sustainable Development Goal 3.1 that seeks to reduce the global maternal mortality ratio to fewer than 70 per 100,000 live births by 2030.

## 1. Background

Political, economic, religious, and social reasons have made pregnancy termination a delicate and divisive reproductive health issue [1]. Induced abortion, miscarriage, and stillbirth are all examples of pregnancy termination [2]. These words have been used interchangeably to describe various forms of pregnancy termination [3,4,5,6,7]. Between 2010 and 2014, nearly 55.7 million pregnancy terminations annually happened in the form of induced abortions worldwide [8]. Despite the fact that induced abortions are legal in some countries, there are regional and sub-regional differences in their prevalence [9]. According to the WHO [10], low-and middle-income countries have more induced abortions than developed countries (35 million versus 77 million), with the likelihood of a woman having an induced abortion being close in both geographical areas (26 induced abortions per 1000 women aged 15–44 years in developed countries versus 29/1000 in low-and middle-income countries [11]. Recent statistics suggest that 56 million induced abortions happen annually worldwide, with a worldwide rate of 29 per 1000 women and approximately half (i.e., over 25 million) being unsafe and performed by unqualified individuals or persons without requisite skills and/or in environments that do not meet appropriate health standards [1,12].

About two million babies die before they are born each year due to stillbirths, with an estimated 18.9 stillbirths per 1000 births annually. By 2015, almost 2.62 million pregnancies had resulted in stillbirths around the world, with developed countries accounting for about 1.8% of these cases and low-and middle-income countries accounting for the remainder. About 98% of stillbirths occur in low- and middle-income countries, equivalent to maternal deaths. Sub-Saharan Africa (SSA) has the highest stillbirth rate in the world, at 28.3 per 1000 births, compared to 3.1 for high-income countries, which accounts for around a third (35.4%) of the global stillbirth burden [13,14]. Stillbirths are rare and mostly not documented in Ghana, just like many other countries in SSA [13]. Stillbirth rates in Ghana have been estimated to range between 14 and 22 per 1000 births [15,16,17], with higher rates recorded in different parts of the country (e.g., 23 stillbirths/1000 births in the Navrongo area of the Upper East region [18], 32 stillbirths/1000 births in the Brong Ahafo region [19], and 59 stillbirths/1000 vaginal deliveries in a tertiary health facility). Data on miscarriages in SSA are quite often not accounted for and overlooked because a significant number of unreported spontaneous pregnancy losses are not seen by health professionals, and hence country-specific estimates are not readily available [20,21,22]. 

In Africa, about 90% of sexually active women of childbearing age lived in countries with restrictive abortion laws as of 2015 [23]. For these nations (e.g., Algeria, Botswana, Gambia, Ghana, Liberia, Mauritius, Namibia, Sierra Leone), a small proportion of women meet the legal conditions to request safe abortion services. These legal restrictions have forced many women to look for services from unqualified sources and/or under insanitary conditions, exposing them to a significant risk of death or disability [24]. As a result, the majority of induced abortions in Africa are considered illegal and unsafe [25], with health complications from unsafe abortions responsible for roughly 14% of all maternal deaths in Africa [9,10]. Unsafe abortions are the leading cause of death in Africa (650 deaths per 100,000) [9]. Despite the fact that many sub-Saharan African countries have stringent abortion laws, Ghana has a relatively liberal abortion law [26]. 

The abortion law, which was passed in 1985, states that an abortion performed by a professional medical practitioner is legal if the pregnancy is induced by rape, incest, or defilement of a “female idiot”; if continuing the pregnancy will endanger the woman’s life or her physical or mental health; or if there is a significant risk that the child will suffer from a severe physical abnormality or disease. Despite the relatively liberal nature of the law in Ghana, unsafe abortions remain a major social and public health issue in the country [27,28,29,30]. According to available data, only 12% of abortions in Ghana are conducted under the guidance of a qualified medical practitioner, with the remaining 88% occurring in unsafe conditions [31]. Due to a lack of or inadequate information regarding safe abortion, stigmatization from unwanted pregnancy, and religious beliefs, many women in Ghana still have unsafe abortions [28,31,32]. In Ghana, about 20% of women of reproductive age (i.e., 15–49 years) have had an induced abortion at some point in their lives [33]. The prevalence of induced abortions ranges from 27 to 61 per 1000 women [34], accounting for 11% of the 350 deaths per 100,000 live births maternal mortality rate in the country. The connection between induced abortion and maternal mortality is especially important because it is a leading cause of death among Ghanaian women [35,36].

To deal with the sexual and reproductive health challenges in Ghana, the government has made efforts to implement comprehensive sexuality education (CSE) programs in schools. However, this aim has not been achieved due to challenges such as insufficient and piecemeal funding for CSE; lack of coordination of the various efforts by central and local government, NGOs and development partners; inadequate systems for monitoring and evaluating teachers and students on CSE; inadequate weight given to CSE when integrated into other subjects; insufficient adaptation of the curriculum to local contexts; and limited stakeholder participation in curriculum development [37].

Previous research has primarily focused on induced abortion in Ghana and other sub-Saharan African states by examining the socio-demographic profile (e.g., age, employment/wealth status, marital status, place of residence (rural or urban), religious affiliation) among sexually active women [31,38,39]. However, most of these country-specific studies in Africa have been restricted by geographical scope and/or been facility-based, often targeting a specific city, region/province, urban/rural areas, or a specific subgroup of women (i.e., young, unmarried, or students) [40,41,42]. Findings from these studies may have not been representative, may be quite outdated, and therefore restrict generalizability to the general population on recent national trends. Additionally, the strength of associations noted in these studies has been inconsistent partly because of the uniqueness of populations and/or contexts on which the studies were conducted. Moreover, there seems to be a dearth of population-based empirical data on the determinants of pregnancy termination in Ghana to redefine sexual and reproductive health policy, and future research.

Therefore, using recent country-specific nationally representative data on determinants of pregnancy termination would provide useful information for important evidence-based country-specific interventions to improve maternal health and to re-align policy directions towards achieving one of the targets of the UN Sustainable Development Goals (i.e., reducing the global maternal mortality ratio to fewer than 70 per 100,000 live births by 2030), and for future research. The present study examined the associations between demographic and socio-economic factors and pregnancy termination among young Ghanaian women.

This paper employs the theory of planned behavior which proposes that individual behavior is determined primarily by the strength of intention to perform that behavior, in order to explain the predictors of pregnancy termination in Ghana. According to the theory, the strength of behavioral intentions is predicted by three variables: attitude towards the behavior; subjective norm, or perceived social pressure to perform the behavior; and perceived behavioral control, or perceptions of the ease or difficulty of performing the behavior [43].

## 2. Materials and Methods

### 2.1. Data Source

**Figure 1:**
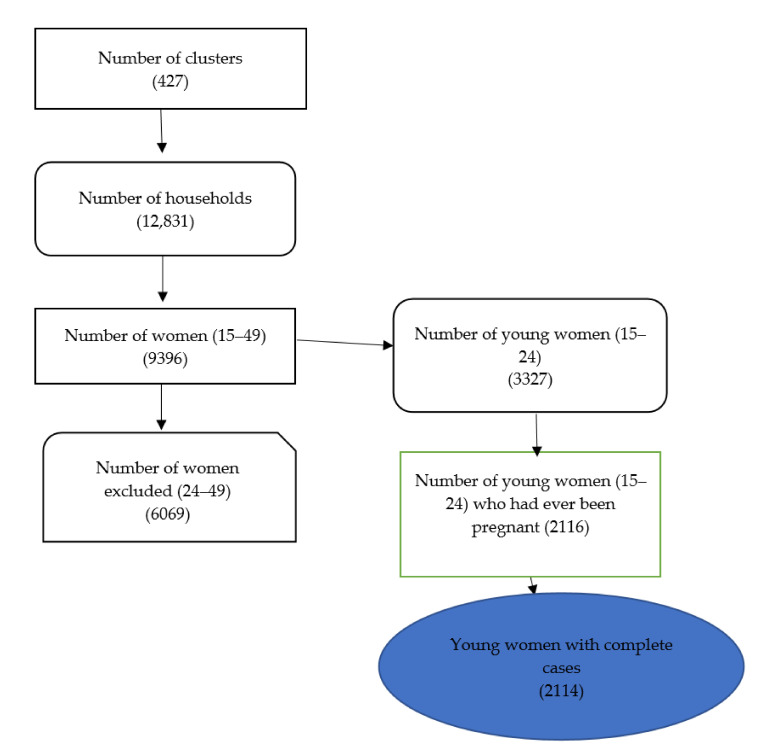
Flow chart showing how the respondents were selected.

Data from Ghana’s 2014 Demographic and Health Survey (DHS) were used for this study. The DHS is conducted in about 85 low- and middle-income countries around the world. Unintended pregnancy, contraception use, qualified birth participation, immunization of under-fives, and intimate partner abuse are among the main maternal and child health issues targeted by the survey. The survey used a stratified two-stage sampling method. The first step was to choose clusters from all over the country, in both urban and rural settings. These were used to establish the study’s enumeration areas (EAs). These clusters were chosen from the country’s ten former administrative regions, spanning urban (*n* = 216) and rural (*n* = 211) areas. The selected EAs were then subjected to a systematic household sampling. A total of 12,831 households were included in the study. A total of 9396 women were interviewed for the survey [44] from the 12,831 households (response rate of 97.3%). Only young women (15–24 years old) who had ever been pregnant and had complete cases on all of the variables studied (*n* = 2114) were included in this analysis. As a result, young women who had never been pregnant (*n* = 1211) were excluded from the study because they had no risk of terminating a pregnancy (see Figure 1).

### 2.2. Study Variables

#### 2.2.1. Dependent Variable

The dependent variable employed for this study was “pregnancy termination” which was derived from the question “have you ever had a terminated pregnancy?”, and the response was coded as 0 = “No” and 1 = “Yes”. 

#### 2.2.2. Explanatory Variables 

Eleven variables were considered as explanatory variables. These are age, wealth quintile, occupation, educational level, religion, marital status, age at first sex, parity, media exposure, place of residence, and region. We selected these variables on the basis that previous studies have found them to have significant associations with pregnancy termination [45,46,47,48,49]. In this study, the original coding for age, wealth quintile, place of residence, and region was maintained, while the remaining six variables were recoded to make them meaningful for the analyses and interpretation of results. Age in the DHS was coded as 15–19 and 20–24 for women aged 15–24. 

### 2.3. Statistical Analyses 

The statistical software Stata version 13 (Stata Corporation, College Station, TX, USA) was used to process the data. All frequency distributions were weighted using the sample weight (v005/1,000,000), while the svy command was used to account for the complex survey design and generalizability of the findings. Both descriptive (frequency, percentages) and inferential (binary logistic regression) analyses were carried out in this study. Statistical significance was pegged at *p* < 0.05. First, frequencies and percentages were used to show the proportion of pregnancy termination across the socio-demographic characteristics of the respondents. This procedure was followed by both bivariate and multivariate binary logistic regression analyses to examine the predictors of pregnancy termination. Model 1 focused on the independent association between each of the explanatory variables and pregnancy termination, while in Model 2, we adjusted for the effect of all the explanatory variables by putting all of them in the same model with pregnancy termination. Binary logistic regression was employed because the dependent variable was measured as a binary factor. Results for the binary logistic regression analyses are presented as crude odds ratios (COR) and adjusted odds ratios (AOR) with their corresponding 95% confidence intervals (CI), signifying the precision and significance of the reported odds ratio values. 

### 2.4. Ethics Approval

Since the data are available in the public domain, no additional approval was needed for this study. However, according to the DHS, ORC Macro Inc.’s Ethics Committee provided ethical clearance. Before starting interviews with each respondent, both respondents gave their informed consent. The authors requested and received permission to download and use the data from MEASURE DHS. The data are available at https://dhsprogram.com/what-we-do/survey/survey-display-437.cfm (accessed on 6 April 2021).

## 3. Results

### 3.1. Descriptive Results on the Prevalence of Pregnancy Termination across Socio-Demographic Characteristics

Table 1 presents the results of the prevalence of pregnancy termination across socio-demographic characteristics of the study participants. The results indicate that young women in Ghana who have had a pregnancy termination are mostly those aged 20–24 (21.7%), those in richer wealth quintile households (22.6%), those working (21.5%), those with primary education (19.2%), traditionalists or those with no religion (22.8%), those who were divorced/separated (29.2%), those whose first sex happened at age 15–19 (18.6%), those with two births (22.1%), those exposed to media (17.4%), urban dwellers (20.5%), and those in the Ashanti region (22.8%). 

### 3.2. Binary Logistic Regression Analysis on the Predictors of Pregnancy Termination

Table 2 presents results on the predictors of pregnancy termination among young women in Ghana. As shown in Model 1 of Table 2, all the explanatory variables had significant associations with pregnancy termination apart from level of education and exposure to media. In the adjusted model (Model 2), age, occupation, marital status, age at first sex, parity, place of residence, and region were found as predictors of pregnancy termination. Specifically, we found that young women aged 20–24 were more likely to have a pregnancy terminated compared to those aged 15–19 (AOR = 3.81, CI = 2.62–5.54). The likelihood of having a pregnancy terminated was high among young women who were working compared to those who were not working (AOR = 1.60, CI = 1.19–2.14). Young women who were married (AOR = 1.92, CI = 1.26–2.93), those cohabiting (AOR = 2.18, CI = 1.43–3.31), and those who were divorced/separated (AOR = 2.17, CI = 1.13–4.18) were more likely to experience a pregnancy termination compared to those who had never married. Young women who had their first sex at the age of 20–24 (AOR = 0.19, CI = 0.10–0.39) and those whose first sex occurred at first union (AOR = 0.57, CI = 0.34–0.96) had lower odds of having a pregnancy terminated compared to those whose first sex happened when they were less than 15 years old. The odds of having a pregnancy terminated reduced with increasing parity with young women with parity of three or more having the lowest odds of having a pregnancy terminated compared to those with no births (AOR = 0.39, CI = 0.21–0.75). The likelihood of pregnancy termination was lower among young women who lived in rural areas (AOR = 0.65, CI = 0.46–0.92) and those in the Upper East region (AOR = 0.18, CI = 0.08–0.39) compared to those in urban areas and the Western region, respectively. 

## 4. Discussion

The current study assessed the predictors of pregnancy termination among young women in Ghana using data from the 2014 GDHS. The results indicate that young women aged 20–24 were more likely to have a pregnancy termination compared to those aged 15–19. This finding is consistent with a study by dos Santos et al. [50]. Several empirical studies in low- and middle-income countries in SSA such as Ghana [51], Kenya [52,53], and Nigeria [54] showed that young women are more likely to have an induced abortion. In Ghana, adolescents are less likely to terminate pregnancies compared to young women because adolescents face barriers such as stigma when accessing abortion services in the country, while a lot of healthcare providers serve as gatekeepers to abortion services provision for adolescents [55,56].

Young women who were working had the highest likelihood of pregnancy termination compared to those who were not working. This finding confirms other studies in Ghana [57], Cameroon [58], and Nigeria [59]. Relatedly, the likelihood of having a pregnancy terminated was high among richer young women compared to the poorest young women. This supports previous studies in Ghana [60,61]. Reasonably, young women who are gainfully employed and from richer households may be financially empowered and can afford to cater for the cost associated with induced abortions compared to their counterparts from poor women households and those who are not working. Hence, women who are poor face more barriers to care and may try to access a variety of traditional or less safe abortion methods [62,63]. 

The odds of pregnancy termination among young women in Ghana were high among those who were married, cohabiting, and divorced/separated compared to those who were never married. Marital status is one of the possible definers of the occurrence or non-occurrence of a pregnancy termination in the existence of an unplanned pregnancy [49]. The association between marital status and pregnancy termination has been documented in studies in Nigeria [63], Nepal [64], and Ghana [46,48,49,57,65]. Specifically, Schwandt et al. [57], Klutsey and Ankomah [39], and Oliveras, Ahiadeke, Adanu, and Hill [65] found that those who sought services were unmarried, divorced, or separated. Nonetheless, this finding contradicts a previous study from about two decades ago which showed that married women were more likely to have an abortion [51]. Perhaps, societal norms that frown on premarital sex as well as childbearing outside wedlock could account for this finding [39]. 

Young women who had their first sex at the age of 20–24 and those whose first sex occurred at first union had lower odds of having a pregnancy terminated compared to those whose first sex happened when they were less than 15 years old. The higher probability of an unintended pregnancy among young women who have sex at earlier ages could be the plausible reason for this finding [66,67]. These unintended pregnancies have been attributed to a lack of or inadequate access to sexual and reproductive health services, including family planning [68,69], as well as the socio-cultural norms surrounding access to contraceptive services among young women having their first sex at an age of less than 15 years [70,71]. One of the means of reducing the barriers to access to family planning services, which increases unintended pregnancies among young women who initiate sex early, is to enhance education through the use of mass media and eliminate all forms of socio-cultural barriers to access sexual and reproductive health services. The likelihood of pregnancy termination, however, increases among young women with no births compared to those with three or more births. This finding suggests that young women who have never experienced pregnancy might not have the readiness for childbirth, especially when they are young and not married, and hence may opt to have their pregnancies terminated [49].

The likelihood of pregnancy termination was lower among young women who lived in rural areas and those in the Upper East region compared to those in urban areas and the Western region, respectively. The low prevalence of pregnancy termination among young women from the rural areas confirms the findings of previous studies in Ghana [46,61], explained by the fact that young women in rural areas are more likely to be poor and unemployed compared to those in urban areas and hence cannot afford to terminate a pregnancy [62,72]. Such women are often in the northern part of Ghana, which includes the Upper East region. Low access to abortion services due to inequalities in the distribution of health facilities between rural and urban areas may be another reason for the lower chances of pregnancy termination among women in rural areas and the Upper East region of Ghana in particular [73,74,75]. In addition, young women from these geographical areas may be deeply entrenched in socio-cultural norms and values that frown on abortions. These young women may prefer giving birth even from unintended pregnancies over terminating them by holding on to their cultural identity.

### Strengths and Limitations

Despite the relatively large sample size, the standard methods employed to ensure the validity and reliability of the data collected and the nationally representative nature of the study make findings generalizable to other homogeneous populations. The study is fraught with some methodological limitations that are worth acknowledging. First, because this was a cross-sectional study, we cannot draw a causal interpretation that these factors are responsible for pregnancy termination among young women in Ghana, and at best, we can only draw associations between the observed outcomes. Second, the study demanded that respondents recall some of the events that had occurred in the past which are subject to recall bias. Additionally, the societal and cultural issues surrounding abortion may influence young people to provide socially desirable responses by under-reporting. For example, it is likely that even those who have ever had a pregnancy terminated might deny ever having done so. Finally, aggregating induced abortions, miscarriages, and stillbirths to derive pregnancy termination, as obtained from the DHS, makes it difficult to understand the predictors of each of these elements of pregnancy termination.

## 5. Conclusions and Policy Implications

Overall, age, occupational status, marital status, age at first sex, parity, place of residence, and region were identified as predictors of pregnancy termination among young women in Ghana. These findings indicate the importance of socio-demographic factors in pregnancy termination. Therefore, government and non-governmental organizations in Ghana should help develop programs (e.g., sexuality education) and strategies (e.g., regular sensitization programs) that reduce unintended pregnancies which often trigger pregnancy termination. These programs and strategies should include easy access to contraceptives that maintains privacy and comprehensive sexual and reproductive health education. Potential interventions should be designed considering the socio-demographic characteristics of young women. Future studies could use longitudinal designs to provide useful insight into the trends and individual/contextual level socio-cultural drivers of pregnancy termination among young women over time in Ghana. Such interventions will help to achieve Sustainable Development Goal 3.1 that seeks to reduce the global maternal mortality ratio to fewer than 70 per 100,000 live births by 2030.

## Figures and Tables

**Table 1:** Prevalence of pregnancy termination across socio-demographic characteristics (*n* = 2114).

Variables	Weighted *n*	Percentage	Pregnancy Termination
**Age**			
15–19	700	33.1	7.7
20–24	1414	66.9	21.7
**Wealth Quintile**			
Poorest	356	16.8	11.4
Poorer	433	20.5	14.8
Middle	521	24.6	17.9
Richer	463	21.9	22.6
Richest	343	16.2	17.2
**Occupation**			
Not working	868	41.1	10.7
Working	1246	58.9	21.5
**Educational level**			
No education	221	10.4	16.7
Primary	380	18.0	19.2
Secondary	1432	67.7	16.5
Higher	82	3.9	17.7
**Religion**			
Christian	1717	81	17.4
Islam	311	14.7	13.9
Traditional/no religion	86	1.3	22.8
**Marital Status**			
Never married	1314	62.2	13.7
Married	351	16.6	20.4
Cohabiting	362	17.1	23.0
Divorced/separated	87	4.1	29.2
**Age at first sex**			
Less than 15 years	304	14.4	17.5
15–19 years	1370	64.8	18.6
20–24 years	188	8.9	7.1
At first union	251	11.9	16.0
**Parity**			
No births	1157	54.7	15.5
One birth	580	27.5	17.6
Two births	264	12.5	22.1
Three or more births	97	4.6	19.2
**Exposure to media**			
No	143	6.8	12.6
Yes	1971	9.3	17.4
**Place of Residence**			
Urban	1022	48.4	20.5
Rural	1092	51.6	13.9
**Region**			
Western	284	13.4	19.8
Central	217	10.3	12.0
Greater Accra	349	16.5	21.5
Volta	168	8.0	14.8
Eastern	225	10.7	16.7
Ashanti	346	16.4	22.8
Brong Ahafo	226	10.7	16.1
Northern	169	8.0	8.7
Upper East	84	4.0	5.1
Upper West	46	2.2	15.1

Source: computed from 2014 GDHS.

**Table 2:** Binary logistic regression analysis on the predictors of pregnancy termination among young women in Ghana.

Variables	Model 1 COR (95% CI)	Model 2 AOR (95% CI)
**Age**		
15–19	Ref	Ref
20–24	3.36 *** (.42–4.66)	3.81 *** (2.62–5.54)
**Wealth Quintile**		
Poorest	Ref	Ref
Poorer	1.28 (0.77–1.81)	0.84 (0.50–1.41)
Middle	1.71 ** (1.17–2.48)	0.92 (0.53–1.59)
Richer	2.40 *** (1.64–3.52)	10.2 (0.56–1.86)
Richest	1.70 * (1.08–2.65)	0.67 (0.33–1.36)
**Occupation**		
Not working	Ref	Ref
Working	1.99 *** (1.54–2.59)	1.60 *** (1.19–2.14)
**Educational level**		
No education	Ref	Ref
Primary	1.41 (0.90–2.19)	1.39 (0.82–2.34)
Secondary	1.09 (0.74–1.62)	0.98 (0.59–1.64)
Higher	1.23 (0.59–2.59)	1.07 (0.44–2.60)
**Religion**		
Christian	Ref	Ref
Islam	0.69 * (0.48–0.97)	0.71 (0.47–1.07)
Traditional/no religion	1.11 (0.62–2.01)	1.07 (0.57–2.01)
**Marital Status**		
Never Married	Ref	Ref
Married	1.42 * (1.04–1.93)	1.92 ** (1.26–2.93)
Cohabiting	2.07 *** (1.51–2.84)	2.18 *** (1.43–3.31)
Divorced/Separated	2.53 ** (1.44–4.41)	2.17 * (1.13–4.18)
**Age at first sex**		
Less than 15 years	Ref	Ref
15–19 years	0.98 (0.69–1.39)	0.69 (0.46–1.04)
20–24 years	0.45 * (0.24–0.84)	0.19 *** (0.10–0.39)
At first union	0.95 (0.61–1.50)	0.57 * (0.34–0.96)
**Parity**		
No births	Ref	Ref
One birth	1.15 (0.86–1.53)	0.64 * (0.44–0.91)
Two births	1.46 * (1.02–2.08)	0.51 ** (0.32–0.81)
Three or more births	1.38 (0.83–2.30)	0.39 ** (0.21–0.75)
**Exposure to media**		
No	Ref	Ref
Yes	1.41 (0.87–2.31)	1.06 (0.60–1.90)
**Place of Residence**		
Urban	Ref	Ref
Rural	0.68 ** (0.53–0.86)	0.65 * (0.46–0.92)
**Region**		
Western	Ref	Ref
Central	0.38 ** (0.22–0.67)	0.35 ** (0.20–0.62)
Greater Accra	0.92 (0.57–1.46)	0.71 (0.43–1.18)
Volta	0.66 (0.39–1.11)	0.67 (0.39–1.16)
Eastern	0.82 (0.52–1.28)	0.75 (0.46–1.21)
Ashanti	1.19 (0.76–1.84)	1.09 (0.69–1.73)
Brong Ahafo	0.61 * (0.39–0.96)	0.61 (0.37–1.01)
Northern	0.35 *** (0.20–0.63)	0.29 *** (0.14–0.59)
Upper East	0.20 *** (0.10–0.40)	0.18 *** (0.08–0.39)
Upper West	0.52 * (0.29–0.92)	0.48 * (0.24–0.95)
pseudo *R*^2^		0.114

Exponentiated coefficients; 95% confidence intervals in brackets. * *p* < 0.05, ** *p* < 0.01, *** *p* < 0.001. Source: computed from 2014 GDHS.

## Data Availability

Dataset and ethical guidelines are publicly available via the following link: https://dhsprogram.com/what-we-do/survey/survey-display-437.cfm, accessed on 1 June 2021.
